# Pharmacogenomics of Atherosclerotic Plaque: Pathophysiological Background and Therapeutic Perspectives

**DOI:** 10.1007/s11883-026-01414-2

**Published:** 2026-05-21

**Authors:** Alfredo Mauriello, Aldo De Falco, Adriana Correra, Antonello D’Andrea, Francesco Giallauria, Antonio Giordano, Vincenzo Russo

**Affiliations:** 1 S.C. Cardiology, Institute National Cancer, IRCCS, Fondazione “G. Pascale”, V. M. Semmola 52, 80131 Naples, Italy; 2 Cardiology Unit, Department of Medical and Translational Sciences, “Monaldi Hospital”, V. L. Bianchi Snc, 80131 Naples, Italy; 3 Cardiology Unit, Department of Cardiology, University of Foggia, 71122 Foggia, Italy; 4 Cardiology and Intensive Care Unit, Department of Cardiology, Umberto I Hospital, Via San Francesco 1, 84014 Nocera Inferiore, Italy; 5 Department of Translational Medical Sciences, “Federico II” University of Naples, Via S. Pansini 5, 80131 Naples, Italy; 6 Sbarro Institute for Cancer Research and Molecular Medicine, Center for Biotechnology, College of Science and Technology, Temple University, Philadelphia, PA 19122 USA

**Keywords:** Pharmacogenomics, Atherosclerosis, Lipid-lowering drugs, Anti-platelet drugs, Dyslipidemia

## Abstract

**Purpose of Review:**

Advances in pharmacogenomics have paved the way for personalized medicine. Cardiovascular diseases still represent the leading cause of mortality worldwide. This review aims to summarize the background, rationale, and evidence of pharmacogenomics in atherosclerosis, in particular, the use of antiplatelet and lipid-lowering drugs.

**Recent Findings:**

Atherosclerotic disease is strongly influenced by the patient’s genetic background. Randomized clinical trials have supported the role of a genotype-guided approach for antiplatelet therapy in patients with coronary heart disease undergoing percutaneous coronary intervention. Furthermore, there is growing evidence to support the association between some genetic variants and poor adherence to statin therapy, for example, due to the development of muscular symptoms. There is evidence for resistance to some drugs for the treatment of dyslipidemia, such as PCSK9 inhibitors.

**Summary:**

Pharmacogenomics can potentially improve patient care by enabling individualized therapy and facilitating the development of novel therapeutic strategies for cardiovascular disease. This is particularly relevant in a field characterized by high morbidity and mortality. These advances may translate into improved clinical outcomes, reduced healthcare costs, and lower cardiovascular mortality.

## Introduction

**Fig. 1:**
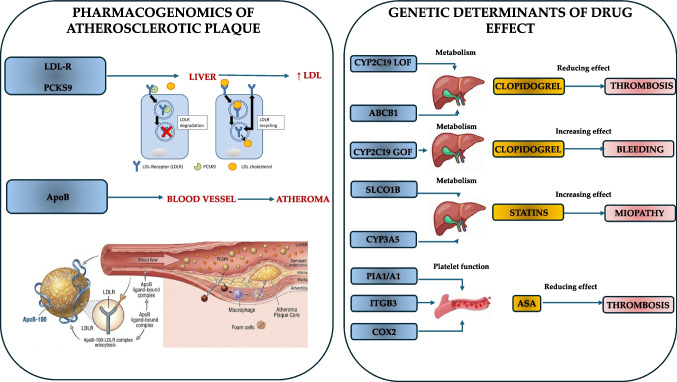
Graphical abstract. The illustration depicts the integration of genetic data into clinical practice to achieve personalized therapy for patients with atherosclerotic cardiovascular disease. ABCB1: ATP binding cassette subfamily B member 1; ApoB: apolipoprotein B; ASA: acetylsalicylic acid; COX-2: cyclooxygenase-2; GOF: gain of function; ITGB3: integrin subunit beta 3; LDL: low-density lipoprotein; LDLR: low-density lipoproteins receptor; LOF: loss of function; PCSK9: proprotein convertase subtilisin/kexin type 9; PLA: phospholipase

The global burden of cardiovascular diseases (CVD) continues to rise, and atherosclerotic vascular diseases are the leading cause of mortality worldwide [1]. The clinical manifestations of atherosclerotic plaque—ranging from stable luminal narrowing to plaque rupture and thrombotic events—reflect a complex interplay between genetic predisposition, environmental exposures, and systemic risk factors such as hyperlipidemia, hypertension, diabetes, and smoking [2, 3]. While conventional risk assessment strategies effectively stratify large populations, a substantial proportion of individuals experience cardiovascular events despite apparently low traditional risk profiles, highlighting the need for deeper insights into the molecular determinants of disease [4]. Genetic factors contribute both to susceptibility to atherosclerosis and to the heterogeneity in therapeutic response. Pharmacogenomics, the study of how genetic variation affects drug response, offers a powerful framework to personalize therapy for atherosclerosis. Variability in genes encoding drug-metabolizing enzymes, transporters, and drug targets can modulate both efficacy and safety of cornerstone therapies. Integrating pharmacogenomic information with conventional risk assessment and polygenic risk stratification has the potential to refine therapeutic strategies, optimize drug selection and dosing, and ultimately improve cardiovascular outcomes [5, 6]. This review aims to provide a comprehensive overview of current evidence linking genetic variation to both the pathophysiology of atherosclerotic plaque and the pharmacological management of atherosclerotic cardiovascular disease. Figure 1 represents the linkage between pharmacogenomics and atherosclerosis.

## Atherosclerosis

Atherosclerosis is a chronic, progressive arterial disease characterized by lipid accumulation, inflammation, calcification, and fibrous remodeling within the intima [7]. The process begins early in life and evolves over decades, driven by complex interactions among hemodynamic forces, metabolic factors, and inflammatory pathways [8]. Atherosclerosis is initiated by the subendothelial retention of oxidized low-density lipoprotein cholesterol (LDL-C), which binds extracellular matrix proteoglycans within the intima [9, 10]. Macrophages and vascular smooth muscle cells (VSMCs) internalize modified LDL-C via scavenger receptors, bypassing physiological feedback regulation of cholesterol uptake and leading to foam cell formation [11]. Notably, this process may occur even at average population levels of LDL-C, contributing to the widespread prevalence of atherosclerosis [12]. Progressive accumulation of lipids, inflammatory cells, and extracellular matrix results in the formation of the atheromatous plaque [10, 13]. Clinical events arise either from progressive luminal narrowing or from plaque rupture. Disruption of the fibrous cap exposes the thrombogenic lipid core, rich in tissue factor (TF), which complexes with factor VIIa and activates the extrinsic coagulation cascade via factors IX and X [14]. Concurrently, von Willebrand factor mediates platelet adhesion to exposed subendothelial collagen through glycoprotein receptors, promoting thrombus formation [15, 16]. Plaque stability is determined by the balance between lipid core expansion and fibrous cap integrity. VSMCs undergo phenotypic switching from a contractile to a synthetic state, producing extracellular matrix components—including collagen, fibronectin, decorin, biglycan, and lumican—that reinforce the fibrous cap [17–19]. In contrast, persistent lipid accumulation promotes macrophage polarization toward a pro-inflammatory M1 phenotype, characterized by cytokine release and protease production, which contribute to necrotic core expansion and cap thinning. Conversely, M2 macrophages exert anti-inflammatory and reparative functions [20, 21]. Systemic inflammation further amplifies atherogenesis. Adipose tissue, particularly in obesity, acts as a source of pro-inflammatory cytokines such as interleukin-6 (IL-6), linking cardiometabolic risk factors to plaque progression and destabilization [11].

## Role of the Endothelium

The vascular endothelium plays a pivotal role in atherogenesis, acting both as a physiological barrier to low-density lipoprotein cholesterol (LDL-C) infiltration and as an active regulator of vascular homeostasis. Endothelial dysfunction represents an early and critical step in disease development and is characterized by impaired nitric oxide (NO) bioavailability and upregulation of leukocyte adhesion molecules, including vascular cell adhesion molecule-1 (VCAM-1) [22, 23]. This dysfunctional phenotype is driven by inflammatory stimuli and hemodynamic alterations, particularly changes in shear stress. Shear stress—the tangential force exerted by blood flow on the endothelial surface—is elevated in regions of laminar flow and reduced at sites of disturbed flow, such as arterial bifurcations, the aortic arch, and the aortic valve. Endothelial cells sense these mechanical forces through shear stress response elements (SSREs), which modulate intracellular signaling pathways and gene transcription. Laminar shear stress promotes an atheroprotective transcriptional program, partly mediated by nuclear factor-κB (NF-κB) signaling and the upregulation of endothelial nitric oxide synthase (eNOS), thereby enhancing NO production and maintaining vascular homeostasis [24]. In contrast, disturbed or low shear stress induces a pro-atherogenic endothelial phenotype, characterized by increased expression of adhesion molecules such as VCAM-1, intercellular adhesion molecule-1 (ICAM-1), and platelet endothelial cell adhesion molecule-1 (PECAM-1), facilitating leukocyte recruitment and vascular inflammation [7].

### Risk Assessment of Atherosclerotic Cardiovascular Disease

Risk stratification for atherosclerotic cardiovascular disease (ASCVD) has traditionally relied on a limited set of established clinical risk factors closely linked to disease pathophysiology. This paradigm, originating from the Framingham Heart Study, is supported by extensive epidemiological evidence and provides the basis for guiding lifestyle modification, pharmacological therapy, and interventional strategies, as well as for monitoring treatment efficacy. The principal determinants include smoking status, arterial hypertension, plasma cholesterol levels, diabetes mellitus, and family history of premature cardiovascular disease [25]. Current guidelines from major scientific societies, including the European Society of Cardiology (ESC) and the American Heart Association (AHA), recommend the use of multivariable risk prediction models—such as SCORE2 and its derivatives—which integrate clinical, biochemical, and, in some cases, instrumental variables to estimate short- and long-term cardiovascular risk [26]. These tools demonstrate robust predictive performance at the population level and are widely adopted due to their practicality and ease of implementation [25, 26]. Nevertheless, a substantial proportion of residual cardiovascular risk remains unaccounted for by conventional risk models. In a cohort of 3,081 patients presenting with ST-segment elevation myocardial infarction (STEMI) (75% male; median age 61 years), 19% had no identifiable traditional risk factors and would therefore not have qualified for preventive interventions under standard risk-based strategies. Importantly, the majority of these individuals (84% of those without risk factors vs. 89% of those with risk factors) exhibited angiographically documented epicardial atherosclerotic disease [27]. These findings underscore the limitations of traditional risk assessment and highlight the need for improved strategies to identify individuals at risk beyond conventional clinical parameters.

### Genetic Basis of Atherosclerosis

Beyond traditional risk factors, genetic determinants significantly contribute to atherosclerotic cardiovascular disease (ASCVD) susceptibility. A family history of premature ASCVD is a well-established independent risk factor, supporting a substantial heritable component in plaque development and progression [25].

The monogenic contribution to atherosclerotic risk is exemplified by familial hypercholesterolemia (FH), an inherited disorder of lipoprotein metabolism characterized by markedly elevated low-density lipoprotein cholesterol (LDL-C) levels and premature ASCVD. FH is most commonly caused by pathogenic variants in the LDL receptor (LDLR) gene, and less frequently by mutations affecting apolipoprotein B (ApoB) or proprotein convertase subtilisin/kexin type 9 (PCSK9), all of which impair hepatic clearance of circulating LDL particles [28].

Heterozygous FH (HeFH) is inherited in an autosomal dominant pattern and has an estimated global prevalence of approximately 1:300, although it remains underdiagnosed. Clinically, it is characterized by persistently elevated LDL-C levels and a family history of premature ASCVD. Residual LDL receptor activity is typically preserved, allowing most patients to respond to lipid-lowering therapies, including statins, ezetimibe, and PCSK9 inhibitors. Genetic testing plays a central role in confirming the diagnosis and enables cascade screening of relatives. Untreated individuals with HeFH carry a 30–50% risk of cardiovascular events by the age of 50, although this risk can be substantially mitigated through early and intensive lipid-lowering strategies [29]. Homozygous FH (HoFH) is a rare and more severe condition resulting from biallelic pathogenic variants that markedly reduce or abolish LDL receptor function. Unlike HeFH, HoFH is characterized by extreme elevations in LDL-C from early childhood, leading to accelerated atherosclerosis and clinical manifestations often occurring in the first or second decade of life. Cutaneous and tendon xanthomas are common. Response to conventional LDL-lowering therapies is frequently attenuated and may vary according to the underlying genotype, particularly in receptor-negative forms. Consequently, many patients require lipoprotein apheresis from an early age to achieve adequate LDL-C control [30].

### Polygenic Risk for Atherosclerosis

Although monogenic disorders such as familial hypercholesterolemia illustrate a clear Mendelian contribution to atherosclerotic risk, they account for only a small fraction of the heritable burden of cardiovascular disease (CVD), which is largely polygenic [31]. Genome-wide association studies (GWAS) have enabled the systematic identification of common genetic variants associated with CVD by comparing allele frequencies between affected individuals and controls. These data can be integrated into polygenic risk scores (PRS), which estimate the cumulative effect of multiple risk and protective alleles within an individual genome [32]. The development of PRS for atherosclerosis is grounded in the “common disease–common variant” hypothesis, whereby highly prevalent conditions are influenced by frequent genetic variants, each conferring modest effect sizes [33]. GWAS typically apply stringent thresholds for statistical significance (P ≈ 5 × 10⁻⁸) to account for multiple testing across millions of loci. However, in polygenic traits, inclusion of variants below this threshold may enhance predictive performance, supporting more inclusive modeling strategies [34]. PRS can be constructed using unweighted or, more commonly, weighted approaches, in which each variant is assigned a coefficient proportional to its effect size derived from GWAS summary statistics [32]. Early PRS models for coronary artery disease (CAD), based on a limited number of lipid-related loci, demonstrated modest predictive ability but did not significantly improve discrimination beyond conventional risk scores [35]. Advances in genotyping and computational methods have enabled the development of genome-wide PRS incorporating millions of variants, substantially improving risk stratification. Contemporary PRS have been shown to predict incident CVD independently of—and incrementally to—traditional risk factors [36], with consistent performance across age groups, thereby addressing an important limitation of conventional risk models in younger individuals [37]. In the GENVASC nested case–control study, addition of a PRS to the QRISK2 algorithm improved correct classification of high-risk individuals by 11.7%, with a 47% improvement among subjects aged 40–54 years [38]. Beyond event prediction, PRS may inform detection of subclinical atherosclerosis. In a prospective cohort of 1,645 patients undergoing coronary computed tomography for suspected stable CAD, a weighted PRS was associated with a higher prevalence of obstructive CAD and increased coronary artery calcium, although not with specific plaque phenotypes [39]. Importantly, elevated genetic risk may identify individuals with adverse prognosis irrespective of angiographic findings. In a cohort of 1,503 patients undergoing coronary angiography, a high genome-wide PRS was associated with increased all-cause mortality, particularly among those without angiographically evident CAD. Incorporation of PRS into conventional risk models significantly improved mortality prediction (AUC 0.70 vs. 0.66; *P* = 0.001) [40]. Current European Society of Cardiology (ESC) consensus statements suggest that PRS assessment may be considered as an adjunct to traditional risk stratification, particularly in younger individuals at intermediate risk, provided that appropriate genetic counseling and informed consent are ensured [41].

## Antiplatelet Drugs

### Clopidogrel

Clopidogrel is an oral, irreversible, second-generation P2Y12 receptor inhibitor widely used in the management of atherosclerotic cardiovascular disease [42]. As a prodrug, approximately 85% is hydrolyzed to an inactive metabolite by hepatic carboxylesterase-1, whereas only ~ 15% undergoes bioactivation through a two-step oxidative process mediated by cytochrome P450 (CYP) enzymes [42, 43]. CYP2C19 plays a central role in both oxidative steps leading to formation of the active thiol metabolite (R-130964) [6].

CYP2C19 is highly polymorphic, with over 25 known allelic variants. The most clinically relevant loss-of-function (LOF) alleles, *2 and *3, result from single-nucleotide polymorphisms that produce truncated, non-functional proteins [44]. Carriers of one LOF allele are classified as intermediate metabolizers, whereas homozygous carriers are poor metabolizers with markedly reduced or absent enzymatic activity. Multiple pharmacokinetic and pharmacodynamic studies consistently demonstrate reduced active metabolite generation and increased high on-treatment platelet reactivity (HPR) among LOF carriers [45–47]. Clinically, these variants are associated with an increased risk of ischemic events compared with non-carriers, prompting interest in genotype-guided antiplatelet strategies [48, 49].

Meta-analyses have confirmed the clinical relevance of CYP2C19 genotype in clopidogrel-treated patients. In a pooled analysis of 9 studies (*n* = 9,685), carriage of one or two LOF alleles was associated with a significantly increased risk of major adverse cardiovascular events (MACE) (HR 1.55 and 1.76, respectively; *p* = 0.002) [50]. A larger meta-analysis including 42,016 patients from 32 studies demonstrated a similar association, although most data were derived from retrospective analyses without genotype-guided treatment allocation [51].

Randomized controlled trials (RCTs) have established the superiority of ticagrelor and prasugrel over clopidogrel in acute coronary syndrome (ACS) [52, 53]. In the PLATO genetic substudy, ticagrelor significantly reduced ischemic events compared with clopidogrel in CYP2C19 LOF carriers (*p* = 0.038), whereas no significant difference was observed among non-carriers [53, 54].

Three major RCTs have evaluated genotype-guided antiplatelet therapy following percutaneous coronary intervention (PCI).

The Italian PHARMCLO trial (*n* = 888) compared a rapid pharmacogenomic-guided strategy—including CYP2C19 (*2, *3, *17) and ABCB1 variants—with standard of care (SOC) in ACS patients [55]. Although prematurely terminated, genotype-guided therapy significantly reduced the composite endpoint of cardiovascular death, myocardial infarction (MI), stroke, and BARC 3–5 bleeding at 12 months (HR 0.58; p ≤ 0.001), driven primarily by fewer ischemic events, with a non-significant trend toward less bleeding.

The POPular Genetics trial (*n* = 2,488 STEMI patients undergoing primary PCI) demonstrated non-inferiority of a CYP2C19-guided strategy (clopidogrel in non-carriers; ticagrelor/prasugrel in LOF carriers) compared with routine use of potent P2Y12 inhibitors [56]. Ischemic outcomes were similar (NACE 5.1% vs. 5.9%; *p* < 0.001 for non-inferiority), while bleeding events were significantly reduced in the genotype-guided arm (9.8% vs. 12.5%; *p* = 0.04).

In TAILOR-PCI (*n* = 5,302), genotype-guided escalation to ticagrelor or prasugrel in LOF carriers showed a non-significant reduction in the primary composite endpoint at 12 months (HR 0.66; *p* = 0.06) without differences in bleeding [57]. Notably, early event reduction at 3 months was significant (HR 0.21; *p* = 0.001), although this benefit attenuated over time.

Evidence supporting genotype-guided therapy extends beyond coronary disease. In a CHANCE substudy of patients with minor stroke or transient ischemic attack (TIA), the benefit of clopidogrel-based dual antiplatelet therapy (DAPT) over aspirin was confined to non-carriers of CYP2C19 LOF alleles (interaction *p* = 0.02) [58]. A subsequent meta-analysis of 4,762 patients with stroke or TIA confirmed a nearly twofold higher risk of recurrent stroke among LOF carriers treated with clopidogrel (RR 1.92; *p* < 0.001).

Data in peripheral artery disease (PAD) remain limited, though observational evidence suggests worse outcomes among LOF carriers [59]. The ongoing GENPAD trial is evaluating a genotype-guided antithrombotic strategy in PAD, including dose escalation of clopidogrel for intermediate metabolizers and dual pathway inhibition (aspirin plus low-dose rivaroxaban) for poor metabolizers [60].

Overall, CYP2C19 genotype significantly influences clopidogrel pharmacokinetics, platelet inhibition, and clinical outcomes. While genotype-guided strategies appear promising—particularly in reducing bleeding without compromising ischemic protection—further large-scale trials are needed to refine their role in routine clinical practice across the spectrum of atherosclerotic disease.

### Ticagrelor and Prasugrel

Pharmacogenomic evidence for ticagrelor and prasugrel remains limited compared with clopidogrel, and most available studies have focused on pharmacodynamic endpoints—primarily platelet reactivity—rather than hard clinical outcomes [54, 61–63]. In the genetic substudy of the PLATO trial, ticagrelor demonstrated consistent efficacy and safety irrespective of CYP2C19 genotype, confirming that its clinical benefit is not influenced by the loss-of-function variants that impair clopidogrel activation [54]. A subsequent two-stage genome-wide association study (GWAS), conducted in approximately one-third of PLATO participants, evaluated whether additional genetic determinants could modulate ticagrelor response [63]. Although variants in SLCO1B1, CYP3A4, and UGT2B7 were associated with differences in plasma concentrations of ticagrelor and its active metabolite (AR-C124910XX), these pharmacokinetic variations did not translate into significant differences in the primary composite clinical endpoint. Overall, current data suggest that common genetic variants do not meaningfully influence clinical outcomes in ticagrelor-treated patients. Similarly, pharmacogenomic data for prasugrel are sparse. In the TRITON-TIMI 38 trial, ABCB1 genotyping was performed in 2,932 patients with acute coronary syndromes undergoing percutaneous coronary intervention (PCI) [62]. ABCB1 polymorphisms were not significantly associated with either pharmacodynamic response or clinical outcomes in prasugrel-treated patients (*P* = 0.129). Consistent findings were reported in a single-center retrospective Japanese study including 1,580 PCI patients stratified by CYP2C19 genotype [61]. In this cohort, the incidence of the composite endpoint (cardiovascular death, myocardial infarction, definite stent thrombosis, ischemic stroke, or major bleeding) did not differ significantly between clopidogrel and prasugrel treatment groups (HR 1.98; 95% CI 0.85–4.61; *P* = 0.12). Taken together, current evidence indicates that, unlike clopidogrel, ticagrelor and prasugrel appear largely unaffected by common pharmacogenetic variants in terms of clinical efficacy and safety, although dedicated prospective pharmacogenomic trials with clinical endpoints remain limited.

### Acetylsalicylic Acid

Acetylsalicylic acid (ASA) exerts its antiplatelet effect through irreversible acetylation of cyclooxygenase (COX-1) (Ser529) and COX-2 (Lys512), thereby inhibiting thromboxane A₂ (TXA₂) synthesis and suppressing platelet aggregation [64]. Despite its well-established efficacy, substantial interindividual variability in platelet response to ASA has been reported [65].

“Aspirin resistance” is a frequently described but heterogeneous phenomenon with multifactorial determinants, including genetic variability. Both candidate gene studies and genome-wide association studies (GWAS) have identified single-nucleotide polymorphisms (SNPs) in genes involved in the COX pathway, TXA₂ biosynthesis, and platelet receptor signaling [66, 67]. However, inconsistent definitions and methodological heterogeneity have limited reproducibility. The European Society of Cardiology (ESC) Working Group on Thrombosis has proposed a distinction between clinical resistance—defined by the occurrence of thrombotic events despite ASA therapy—and laboratory resistance, characterized by inadequate platelet inhibition on functional testing [68].

Among candidate genes, PTGS1 and PTGS2, encoding COX-1 and COX-2, have been extensively investigated. Variants such as rs10306114 and rs3842787 in PTGS1 have been associated with impaired platelet response, although findings remain inconsistent across studies [69, 70]. PTGS2 is highly polymorphic, but only a limited number of variants appear to influence enzyme expression or activity [71, 72]. The rs20417 C allele has been associated with aspirin resistance in patients with ischemic stroke (adjusted OR 1.75; 95% CI 1.06–2.88; *p* = 0.016; higher risk in GC and CC genotypes), although these findings have not been uniformly replicated [73].

Genetic variation in platelet receptor genes has also been implicated. Polymorphisms in ITGB3, encoding glycoprotein IIb/IIIa, have been associated with altered aspirin responsiveness and thrombotic risk [74]. In a study of 80 healthy subjects, carriers of the PlA2 allele (rs5918) exhibited significantly reduced aspirin sensitivity compared with wild-type individuals (*p* = 0.001) [74]. A subsequent systematic review of 10 studies confirmed an association between the PlA2 allele and laboratory aspirin resistance in healthy adults (OR 2.36; 95% CI 1.24–4.48; *p* = 0.009) [75].

However, translation into clinical outcomes remains uncertain. In a post hoc pharmacogenetic analysis of the ASPREE trial, which randomized 1,486 healthy elderly (> 70 years old) individuals to 100 mg daily ASA or placebo, no significant interaction was observed between rs12041331 genotype and MACE or bleeding outcomes (*P* = 0.13) [76, 77]. In the same trial, the carriers of rs3798220-C lipoprotein (a) [Lp(a)] SNP, associated with elevated plasma of Lp(a), had a greater than 50% reduction in MACE compared to those randomized to placebo (HR 0.44, 95% CI 0.20–0.94, *p* = 0.033). However, the non-carriers randomized to aspirin did not exhibit a significant risk reduction (HR 0.91, 95% CI 0.77–1.08, *p* = 0.30) [78].

Overall, although several genetic variants have been associated with laboratory measures of aspirin responsiveness, their impact on clinical endpoints remains inconsistent, and routine pharmacogenetic testing for ASA therapy is not currently supported by robust evidence.

## Lipid-Lowering Drugs

### Statins

Statins, competitive inhibitors of 3-hydroxy-3-methylglutaryl coenzyme A (HMG-CoA) reductase, represent the cornerstone of LDL-cholesterol (LDL-C) lowering therapy in both primary and secondary prevention of coronary artery disease (CAD) [79, 80]. However, marked interindividual variability exists in lipid-lowering efficacy and in the risk of adverse events, particularly statin-associated muscle symptoms (SAMS). Pharmacogenetic studies have therefore focused on genes involved in statin pharmacodynamics and pharmacokinetics.

Polymorphisms in the HMGCR gene may influence statin responsiveness. The intronic variant rs3846662 modulates alternative splicing of exon 13, resulting in expression of a truncated HMGCR isoform (HMGCRv1) lacking part of the catalytic domain targeted by statins [81]. Although some studies have linked this variant to attenuated LDL-C reduction, findings remain inconsistent [82]. Similarly, the KIF6 rs20455 variant was initially proposed as a predictor of cardiovascular risk and statin benefit, but subsequent analyses failed to confirm clinical utility, and routine testing is not recommended [81].

Greater clinical relevance has been demonstrated for variants in SLCO1B1, encoding the hepatic uptake transporter OATP1B1. The c.521 T > C (rs4149056) polymorphism, defining the SLCO1B1*5 and *15 haplotypes, reduces transporter activity, leading to increased plasma statin concentrations—particularly simvastatin—and a higher risk of myopathy [82–84]. In genome-wide analyses, this variant accounted for a substantial proportion of simvastatin-induced myopathy cases, with heterozygous carriers exhibiting an approximately 4.5-fold increased risk [82]. Consequently, high-dose simvastatin should be avoided in individuals with reduced-function SLCO1B1 variants.

Variants affecting statin efflux transporters are also clinically relevant. The ABCG2 rs2231142 polymorphism reduces transporter activity and increases systemic exposure to rosuvastatin. Clinical Pharmacogenetics Implementation Consortium (CPIC) guidelines recommend initiating rosuvastatin at lower doses (e.g., 20 mg maximum starting dose) in individuals with reduced-function alleles, with consideration of alternative or combination therapy if further LDL-C lowering is required [85].

Cytochrome P450 enzymes further contribute to statin pharmacokinetic variability. The decreased-function CYP3A4*2 allele (rs35599367) significantly reduces CYP3A4 activity and increases exposure to statins metabolized by this pathway, including simvastatin, atorvastatin, and lovastatin [86–88]. CYP3A5*3 (rs776746), a loss-of-function variant, has also been associated with increased simvastatin exposure, with up to a threefold higher area under the curve (AUC) in homozygous carriers compared with wild-type individuals [84, 89–91].

Fluvastatin metabolism is primarily mediated by CYP2C9. Reduced-function alleles, including CYP2C9*2 (rs1799853) and CYP2C9*3 (rs1057910), significantly increase systemic exposure. Homozygous CYP2C9*3 carriers may exhibit up to a threefold increase in fluvastatin AUC [92, 93], and enhanced LDL-C reduction has been observed in some reduced-function genotypes [88]. These variants are incorporated into the 2022 CPIC guidelines, which recommend lower starting doses (≤ 20 mg/day) and dose adjustment in CYP2C9 poor metabolizers [85].

Overall, pharmacogenetic variability in hepatic transporters and metabolizing enzymes—particularly SLCO1B1 and CYP2C9—has clinically meaningful implications for statin safety, whereas genetic determinants of LDL-C lowering efficacy remain less consistently validated.

### Ezetimibe

Ezetimibe is a selective inhibitor of intestinal cholesterol absorption that targets the Niemann-Pick C1-like 1 (NPC1L1) sterol transporter on enterocytes [94, 95]. NPC1L1 mediates uptake of both dietary cholesterol and plant sterols, and its inhibition reduces plasma LDL-C levels. Following absorption, ezetimibe undergoes extensive phase II metabolism via phenolic glucuronidation within the intestinal wall, with over 95% circulating as ezetimibe-glucuronide upon entry into the portal vein [95]. The liver subsequently excretes the drug into bile, facilitating enterohepatic recirculation and prolonged intestinal activity. Importantly, ezetimibe is not metabolized by cytochrome P450 enzymes, minimizing the risk of drug-drug interactions [95].

Hepatic uptake of ezetimibe-glucuronide is mediated primarily by the OATP1B1 transporter (SLCO1B1). Polymorphisms in SLCO1B1, such as c.388A > G, can alter transporter substrate recognition and influence hepatic drug disposition, though the pharmacodynamic impact appears minimal [96, 97]. In enterocytes, ezetimibe glucuronidation is catalyzed by UDP-glucuronosyltransferase 1A1 (UGT1A1), and both the parent drug and glucuronide are effluxed via ABCB1 and ABCC2 transporters [93]. Induction of these enzymes and transporters—for example, by rifampin—can accelerate ezetimibe clearance and abolish its LDL-C-lowering effect [98]. Functional genetic variants in ABCB1, ABCC2, and UGT1A1 may modulate drug exposure, with reduced-function alleles potentially enhancing efficacy and increased-function alleles diminishing response, although clinical evidence remains limited [93].

Genetic variation in NPC1L1 itself has been associated with differential LDL-C lowering. Rare compound heterozygous variants (V55L in exon 2 and t3754a in exon 18) have been linked to non-response to ezetimibe in individual cases [99, 100]. In larger cohorts, carriers of non-reference haplotypes—including the absence of the common 1735C-25342A-27677 T haplotype or the presence of the −133A, −18A, 1679G combination—exhibited greater reductions in LDL-C compared with reference haplotype carriers [101, 102]. Overall, the pharmacogenomics of ezetimibe suggests that variation in NPC1L1, as well as genes encoding metabolizing enzymes and transporters (UGT1A1, ABCB1, ABCC2, SLCO1B1), can influence drug disposition and, to a lesser extent, clinical response. While some variants appear to enhance LDL-C lowering, the clinical significance of these genetic differences remains to be fully elucidated.

### Proprotein Convertase Subtilisin/Kexin Type 9 (PCSK9) Inhibitors

PCSK9 inhibitors target a serine protease that regulates plasma LDL-C levels by promoting degradation of LDL receptors through binding to the EGF-A domain [103]. Reduced LDL receptor availability decreases LDL-C clearance, whereas monoclonal antibodies against PCSK9 can lower LDL-C by up to 57% as monotherapy and up to 73% when combined with statins [104, 105].

Despite extensive data on PCSK9 mutations and their impact on LDL-C, pharmacogenomic determinants of response to PCSK9 inhibitors remain poorly understood. Case reports in patients with familial hypercholesterolemia illustrate that mutations in LDLR can attenuate therapeutic response. For example, a heterozygous LDLR W483X mutation was associated with suboptimal LDL-C reduction during PCSK9 inhibitor therapy, likely due to impaired LDL receptor metabolism [106]. In another case, compound heterozygous LDLR mutations (R410S and G592E) were linked to reduced response despite maximal therapy with statin, ezetimibe, and PCSK9 inhibition [107]. Functional analyses suggested that LDLR-G592E (class 2b) causes defective ER exit and receptor degradation, while LDLR-R410S modulates PCSK9-mediated receptor degradation in endosomes/lysosomes. These findings highlight how specific combinations of LDLR mutations may interfere with expected PCSK9 inhibitor efficacy. In addition, a PCSK9 gene duplication, described in a case report, may be a potential mechanism underlying nonresponse to PCSK9 inhibition [108].

Currently, there are no large-scale studies evaluating the impact of PCSK9 gene variants on therapeutic response. Pharmacogenomics offers a promising approach to identify patients who may exhibit suboptimal response to PCSK9 inhibitors and could guide personalized therapy.

### Inclisiran

Inclisiran is a novel lipid-lowering therapy that employs RNA interference (RNAi) to selectively silence hepatic PCSK9 expression, thereby enhancing LDL receptor recycling and markedly reducing LDL-cholesterol (LDL-C) levels. Unlike monoclonal antibodies targeting PCSK9, inclisiran is a small interfering RNA (siRNA) conjugated to a triantennary N-acetylgalactosamine (GalNAc) moiety, which enables targeted hepatocyte uptake via the asialoglycoprotein receptor [109]. This mechanism allows for infrequent dosing—every six months—while achieving sustained LDL-C reductions of approximately 50% when added to maximally tolerated statin therapy [110].

The pharmacogenomic profile of inclisiran remains largely unexplored. Given its post-transcriptional mechanism, genetic variation in traditional drug-metabolizing enzymes or transporters (e.g., CYP450) is unlikely to influence pharmacokinetics or pharmacodynamics. However, genetic polymorphisms in the PCSK9 gene or in pathways affecting RNAi machinery and hepatic uptake—such as the asialoglycoprotein receptor (ASGR1/ASGR2)—may theoretically modulate drug efficacy, although clinical evidence is currently lacking. Post-hoc analyses from the ORION-9, ORION-10 [111], and ORION-11 [112] trials have not identified clinically meaningful genotype–response associations, suggesting that inclisiran provides consistent LDL-C reduction across diverse populations.

### Fibric Acid Derivatives (Fibrates)

Fibrates are amphipathic carboxylic acids that reduce plasma triglycerides, reduce very-low-density lipoprotein (VLDL) levels and increase HDL-C levels, primarily through activation of peroxisome proliferator-activated receptor alpha (PPAR-α) and subsequent transcriptional modulation of lipid metabolism genes [113, 114]. Pharmacogenomic studies have explored genetic determinants of interindividual variability in fibrate response. Variants in the APOA1/C3/A4/A5 cluster, together with polymorphisms in LFABP, LIPC, ABCG8, and FABP1, collectively account for ~ 20% of the observed variation in triglyceride and HDL-C responses [115, 116]. Rare variant analyses have identified additional loci in PPARA, LPL, and APOC3 that influence fibrate efficacy. Genome-wide association studies (GWAS) from the GOLDN and ACCORD trials have further implicated PBX4, SMAD3, and IPO11, while rare variants in AKR7A3, HSD17B13, ITGA7, SIPA1L2, and CEP72 were associated with enhanced fibrate responses [117]. Among common variants, rs964184 near the APOA1 gene has emerged as a consistent predictor of fenofibrate response, demonstrating significant effects on HDL-C and triglycerides, with a trend toward LDL-C modulation [118]. Despite these promising findings, the clinical utility of pharmacogenomic-guided fibrate therapy remains limited, as replication and functional validation are required before these variants can inform treatment decisions. Table 1 summarizes major classes of cardiovascular drugs and the genes that might affect drug response*.*

**Table 1:** Major classes of cardiovascular drugs and the genes that might affect drug response

Pharmacogenomics of major cardiovascular drugs			
Drug	Genes	Effect and mechanism	
Acetylsalicylic acid	ITGB3	Acetylsalicylic acid resistance	Possible increased thrombotic risk
	PIA1/A2		
	PTGS1 (COX1)		
	PTGS2 (COX2)		
Clopidogrel	CYP2C19*2 and *3	LOF alleles can reduce the formation of the active metabolite	Increased thrombotic risk
	CYP2C19*17	GOF allele	Increased haemorrhagic risk
	ABCB1	Reduced intestinal absorption of clopidogrel	Increased thrombotic risk
Ticagrelor and Prasugrel	CYP3A4	Pharmacokinetic difference without clinical impact	
	UGT2B7		
	SLCO1B1		
	CYP2C19		
Statins	HMG-CoA reductase	Reduced statin binding	LDL reduction less than expected
	SLCO1B1*5 and *15	Reduce hepatic uptake, increased plasma level	Higher toxicity risk
	*CYP3A5*	Slower inactivation	Higher toxicity risk
Ezetimibe	*SLCO1B1* c388A > G variant	Increased enterohepatic circulation of the drug	No effect on drug response
	ABCB1, ABCC2, and UGT1A1	Various mutations can increase or decrease drug clearance	Variable effect
PCSK9-i	LDLR	Reduced LDL-r expression regardless of PCSK9 function	Reduced effect, especially in HoFH
Fibrates	APOA1, CETP	Increased drug response	

*ABCB1* ATP binding cassette B1; *ABCC2* ATP-binding cassette C2; *APOA1* Apolipoprotein A1; *CYP2C19* Cytocrome P450 2C19; *CYP3A4* Cytocrome P450 3A4; *CYP3A5* Cytocrome P450 3A5; *HMG-CoAreductase* 3-hydroxy-3-methylglutaryl-coenzyme A reductase; *LDLR* Low-density lipoprotein receptor; *PCSK9-i* Proprotein convertase subtilisin/kexin type 9; *PIA1/A2*: Glycoprotein Iib/IIIA allele; *PTGS1* prostaglandin-endoperoxide synthase 1; *PTGS2* prostaglandin-endoperoxide synthase 2; *SLCO1B1* solute carrier organic anion transporter family member 1B1; *UGT2B7* UDP-glucuronosyltransferase Family 2 Member B7

## Poligenic Influence

The role of endothelial cell (EC) dysfunction in contributing to an individual’s susceptibility to coronary atherosclerosis, and the manner in which LDL-C concentrations appear to modulate this relationship.

Marston et al. [119] identified variants with effects on EC function and constructed a 35 SNP polygenic risk score comprising these EC-specific variants (EC PRS) and the genes encoding proteins of the nitric oxide pathway. The association of the EC PRS with the risk of incident CVD was tested in three cohorts: a primary prevention population in the UK Biobank (UKBB; *n* = 348,967); a primary prevention cohort from a trial that tested a statin [Use of Statins in Primary Prevention: An Intervention Trial Evaluating Rosuvastatin (JUPITER), *n* = 8,749]; and a secondary prevention cohort that tested a PCSK9 inhibitor (Further Cardiovascular Outcomes Research With PCSK9 Inhibition in Subjects With Elevated Risk (FOURIER), *n* = 14,298). As the first result, in the UKBB, the EC PRS was independently associated with the risk of incident CAD (adjusted HR per 1 s.d. of 1.24 (95% CI 1.21–1.26), *p* < 2 × 10^–16^). This suggests that the functional integrity of the vascular wall is just as important as circulating lipid levels in determining the onset of the disease.

In addition, the clinical benefit of LDL-C lowering was significantly greater in individuals with a high EC PRS than in individuals with low or intermediate EC PRS, with relative risk reductions of 68% (HR 0.32) versus 29% (HR 0.71) in the primary prevention cohort (p= 0.02) and 33% (HR 0.67) versus 8% (HR 0.92) in the secondary prevention cohort (p = 0.01).

Mega et al. [120] analyzed primary and secondary prevention trials. The authors evaluated a community-based cohort study (the Malmo Diet and Cancer Study) and four RCTs of both primary prevention [JUPITER and Anglo-Scandinavian Cardiac Outcomes Trial (ASCOT)] and secondary prevention [Cholesterol and Recurrent Events (CARE) and Pravastatin or Atorvastatin Evaluation and Infection Therapy–Thrombolysis in Myocardial Infarction 22 (PROVE IT-TIMI 22)] with statin therapy, including a total of 48,421 patients. Patients were divided into low (quintile 1), intermediate (quintiles 2–4), and high (quintile 5) genetic risk categories. A genetic risk score identified individuals at increased risk for both incident events (intermediate risk aHR 1.31 (CI 95%, 1.19–1.45, *p* < 0.0001) versus high-risk aHR 1.72 (CI 95%, 1.53–1.92, *p* < 0.0001), respectively) and recurrent coronary heart disease events (intermediate risk aHR 1.65 (CI 95%, 1.19–2.30, *p* = 0.003) versus high-risk aHR 1.81 (CI 95%, 1.22–2.67, *p* = 0.0029), respectively). Individuals with the highest burden of genetic risk derived the greatest relative and absolute clinical benefit from statin therapy (34%, 32%, and 50%, respectively, in the primary prevention trials and 3%, 28%, and 47% in the secondary prevention trials; p = 0.028 ), resulting in a roughly threefold decrease in the number needed to treat to prevent one coronary heart disease event in the primary prevention trials.

A consistent finding across these two studies [119] is that the genetic risk score not only predicts adverse events but also identifies the individuals who derive the greatest clinical benefit from lipid-lowering drugs. Individuals at high genetic risk demonstrate significantly greater risk reductions compared to those at low risk, and the NNT to prevent an event decreases by up to threefold in the context of primary prevention.

Marston et al. in a sub-analysis of the FOURIER Trial, aimed to evaluate the ability of a genetic risk score to predict risk in established cardiovascular disease and identify individuals who derive greater benefit from PCSK9. They included a27-SNP genetic risk score, which defined low (quintile 1), intermediate (quintiles 2–4), and high (quintile 5) genetic risk. Patients were also categorized by major atherosclerotic risk factors, including diabetes mellitus, hypertension, low-density lipoprotein cholesterol ≥ 100 mg/dl, and smoking; multiple (≥ 2) risk factors were considered high clinical risk. After adjusting for clinical factors, during a follow-up of 2.3 years, the genetic risk score was associated with risk for both MACE (p for trend = 0.005) and major coronary events (p for trend < 0.0001). There was no benefit for MACE in patients without multiple clinical risk factors or high genetic risk (HR, 1.02; absolute risk reduction [ARR], −0.2%, *P* = 0.86). In contrast, there was a 13% relative risk reduction (HR, 0.87 [0.75–0.998], *p* = 0.047) and a 1.4% ARR in patients with multiple clinical risk factors but without high genetic risk and a 31% relative risk reduction (HR, 0.69 [0.55–0.86], *p* = 0.0012), and 4.0% ARR in patients with high genetic risk, irrespective of clinical risk (*p* for trend for HR = 0.017, *p* for trend for ARR *p* = 0.004). Patients with high genetic risk who received evolocumab had event rates similar to those of patients with a low burden of both genetic and clinical risk (HR, 1.02, ARR, –0.2%, *p* = 0.86).

Analysis of the FOURIER trial highlights that patients with high genetic risk derive the greatest benefit from treatment with evolocumab, regardless of the presence of other clinical risk factors. Conversely, patients with low genetic risk and few clinical risk factors appear to derive minimal or no benefit from intensive treatment in terms of MACE reduction.

In conclusion, intensive pharmacological treatment is capable of compensating for genetic disadvantage: treated patients with high genetic risk achieve event rates similar to those of subjects with low baseline risk, effectively neutralizing their congenital predisposition.

## Conclusions

Pharmacogenomics may offer a transformative lens for understanding and managing atherosclerotic cardiovascular diseases. Current evidence suggests that genetic variation can profoundly influence both the development of atherosclerotic plaques and individual responses to key pharmacotherapies. Variants in drug-metabolizing enzymes, transporters, and drug targets have been consistently associated with differences in efficacy, safety, and adverse event profiles, underscoring the potential for personalized therapy. Despite significant progress, several challenges remain. Most pharmacogenomic data derive from candidate-gene or post-hoc analyses, with limited prospective, genotype-guided clinical trials. Furthermore, polygenic risk scores and rare variant analyses have revealed the complex, multifactorial nature of atherosclerotic risk, but translating these findings into actionable clinical strategies remains in its infancy. Integration of pharmacogenomic data with conventional risk assessment, imaging biomarkers, and circulating biomarkers may enhance risk stratification, optimize therapeutic selection, and reduce adverse events. Future research should focus on large-scale, multi-ethnic, prospective studies to validate existing genetic associations and evaluate the clinical utility of genotype-guided interventions. Advances in high-throughput sequencing, multi-omics approaches, and machine learning algorithms are likely to uncover novel variants and predictive models, enabling precision medicine in cardiovascular care. Ultimately, the incorporation of pharmacogenomic insights into routine clinical practice holds the promise of improving cardiovascular outcomes by tailoring therapy to individual’s genetic profile, moving beyond the “one-size-fits-all” paradigm of current atherosclerosis management.

## Data Availability

No datasets were generated or analysed during the current study.
